# DCB-3503, a Tylophorine Analog, Inhibits Protein Synthesis through a Novel Mechanism

**DOI:** 10.1371/journal.pone.0011607

**Published:** 2010-07-15

**Authors:** Ying Wang, Wenli Gao, Yuri V. Svitkin, Annie Pei-Chun Chen, Yung-Chi Cheng

**Affiliations:** 1 Department of Pharmacology, Yale University School of Medicine, New Haven, Connecticut, United States of America; 2 Department of Biochemistry and Goodman Cancer Center, McGill University, Montreal, Quebec, Canada; National Cancer Institute, United States of America

## Abstract

**Background:**

DCB-3503, a tylophorine analog, inhibits the growth of PANC-1 (human pancreatic ductal cancer cell line) and HepG2 (human hepatocellular cancer cell line) tumor xenografts in nude mice. The inhibition of growth leads to cancer cell differentiation instead of cell death. However, the mechanisms of action of tylophorine analogs is unknown.

**Methodology/Principal Findings:**

In this study, we show that DCB-3503 suppresses the expression of pro-oncogenic or pro-survival proteins with short half-lives, including cyclin D1, survivin, β-catenin, p53, and p21, without decreasing their mRNA levels. Proteasome inhibitor reversed the inhibitory effect of DCB-3503 on expression of these proteins. DCB-3503 inhibited the incorporation of radiolabeled amino acid and thymidine, and to a much lesser degree of uridine, in a panel of cell lines. The mechanism of inhibition of protein synthesis is different from that of cycloheximide (CHX) as assayed in cell culture and HeLa *in vitro* translation system. Furthermore, in contrast to rapamycin, DCB-3503 does not affect protein synthesis through the mTOR pathway. DCB-3503 treatment shifts the sedimentation profiles of ribosomes and mRNAs towards the polysomal fractions while diminishing monosome abundance, indicative of the inhibition of the elongation step of protein synthesis. Preferential down regulation of several studied proteins under these conditions is likely due to the relative short half-lives of these proteins.

**Conclusion/Significance:**

The inhibitory effect of DCB-3503 on translation is apparently distinct from any of the current anticancer compounds targeting protein synthesis. Translation inhibitors with novel mechanism could complement current chemotherapeutic agents for the treatment of human cancers and suppress the occurrence of drug resistance.

## Introduction

**Figure 1:**
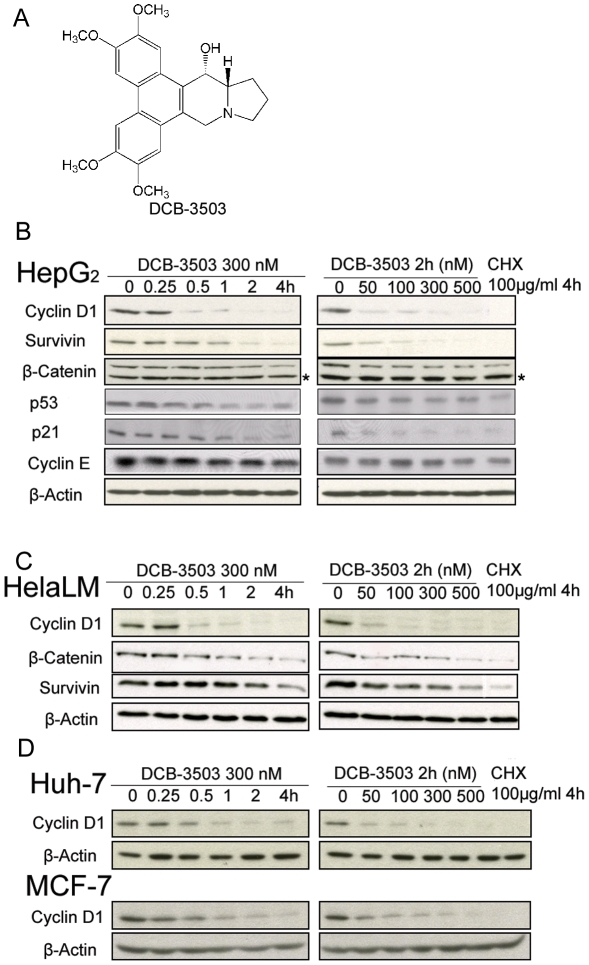
DCB-3503 down regulated expression of cyclin D1, survivin, β-catenin, p53, and p21. (**A**) Structure of DCB-3503. (**B**) and (**C**) HepG2 cells and HeLa cells were treated with DCB-3503 for the indicated time and dose. Whole cell lysates were probed with antibodies as indicated. β-Actin blot was used as internal loading control. **D.** Down-regulation of cyclin D1 by DCB-3503 in Huh7 and MCF-7 cells. **B**, **C**, and **D** are representatives from at least three separate experiments. (* Indicating non-specific band).

Tylophorine analogs are phenanthroindolizidine alkaloids, their pharmacological actions were first described in 1935 [1]. Studies from the 1970's showed that tylocrebrine, tylophorine, and cryptopelurine inhibit protein synthesis, and inhibit RNA and DNA synthesis to a lesser extent [2], [3]. Since then, tylophorine analogs have been the subject of numerous investigations due to their potent antitumor activity [2], [4]. Evaluation of (+)-(*S*)-tylophorine [DCB-3500 (NSC-717335)] and its analog DCB-3503 (NSC-716802) (Fig. 1A) in the antitumor screen at the National Cancer Institute (NCI) showed a fairly consistent and potent growth inhibitory activity (GI_50_ ∼10^−8^ M) in all 60 cancer cell lines, with notable selectivity toward several cell lines, such as melanoma and lung tumor cell lines [5]. Furthermore, NCI's COMPARE program indicated that their activity was unique among known antitumor compounds, suggesting a mode of action that is different from current cancer chemotherapeutic compounds [5]. DCB-3503 has suppressive activity against HepG2 and PANC-1 tumor xenografts in nude mice [5], [6]. It could also decrease clonogenic activity of HepG2 and PANC-1 cells [5], [6], [7]. In addition, DCB-3503 is active against chemotherapeutic resistant KB cell lines overexpressing multidrug resistance (MDR) and multidrug resistance-associate protein (MRP), and Topo I down-regulated cells in cell culture [5].

The details of the mechanisms of action of tylophorine analogs against cancer remain largely unknown [1]. Our previous structure-activity relationship studies revealed that these analogs would have the same mechanism of action [8]. The most potent analog, DCB-3503, had a potent inhibitory effect on NF-κB and less effect on cyclic AMP response elements (CREs) and activator protein-1 sites (AP-1)-mediated transcriptions [5]. DCB-3503 could also down-regulate a subset of proteins, including cyclin D1 [6]. In the present study, we chose several pro-oncogenic and pro-survival proteins, including cyclin D1, survivin, β-catenin, p53, and p21 as model proteins to examine the inhibition effects of DCB-3503 on translation, particularly focusing on cyclin D1. House keeping gene products like β-actin and tubulin are encoded by mRNAs with short and unstructured untranslated regions (UTRs) that are rarely modified. While proto-oncoproteins like cyclin D1 and pro-survival factors like survivin are encoded by mRNAs with long and highly structured UTRs that can be controlled at multiple levels. For example, the expression of cyclin D1, survivin, and β-catenin can be translationally regulated through the AU rich elements in the 3'UTR [9]. Cyclin D1 expression can also be controlled by the PI3K/Akt/mTOR/p70S6K signaling pathways [10]. Understanding the mode of regulatory effect of DCB-3503 on these proteins could help reveal the mechanisms of action of tylophorine analogs.

The current study revealed a novel activity of DCB-3503, which is directed against synthesis of a subset of cell regulatory proteins. This mode of action is distinct from any other known anticancer drugs. Based on our current findings and previous reports of the unique structures, mode of actions, anticancer activities independent of MDR-1 and MRP-1, tylophorine analogs have great potential as a new class of anticancer drugs.

## Materials and Methods

### Materials

Tylophorine analog DCB-3503 (NSC-716802) was synthesized in Dr. D. C. Baker's laboratory (The University of Tennessee, TN). Cell culture media, fetal bovine serum (FBS) were purchased from Invitrogen (Carlsbad, CA). All chemicals except otherwise noted were purchased from Sigma-Aldrich (St. Louis, MO). [^3^H]-amino acid mixture was from GE Healthcare (Arlington Heights, IL). [^14^C]-thymidine was from Moravek (Brea, CA). [^14^C]-uridine was from MP Biomedicals (Solon, OH). EXPRE^35^S^35^S protein labeling mix was from Perkin Elmer (Waltham, MA). Nuclease S7 was from Roche (Indianapolis, IN). Creatine phosphate and MG-132 were from Calbiochem (Gibbstown, NJ). 19 unlabelled amino acid mix (lacking L-methionine) was from Promega (Madison, WI). Rapamycin was from Cell Signaling Technology (Danvers, MA).

### Cell lines and growth conditions

All cell lines were obtained from the American Type Culture Collection (ATCC). HepG2, Huh7, and MCF-7 cells were maintained in RPMI 1640 medium supplemented with 10% FBS. HeLa and PANC-1 cells were maintained in DMEM containing 4.5 g/l glucose supplemented with 10% FBS. All cell lines were maintained in a humidified incubator with an atmosphere of 95% air and 5% CO_2_ at 37°C.

### Western blot analysis

Western blot analysis was done using primary antibodies against cyclin D1 (Cell Signaling Technology), cyclin E (Santa Cruz Biotechnology, Santa Cruz, CA), β-catenin (Cell Signaling Technology), survivin (Cell Signaling Technology), p53 (Santa Cruz Biotechnology), p21 (Santa Cruz Biotechnology), phospho-p70 S6 kinase (Thr^389^) (Cell Signaling Technology), phospho-S6 ribosomal protein (Ser^235/236^) (Cell Signaling Technology), phospho-4E-BP1 (Ser^65^) (Cell Signaling Technology), phospho-4E-BP1 (Thr^37/46^) (Cell Signaling Technology), 4E-BP1 (Cell Signaling Technology), and β-actin (Sigma-Aldrich) at optimal dilution.

### RNA isolation, real-time PCR and Northern blot analysis

Total cellular RNA was isolated using the RNeasy kit (Qiagen, Valencia, CA) for real-time PCR and Northern blot analysis. Poly(A) mRNA was isolated using the Oligotex™ mRNA kit (Qiagen). Real-time PCR was performed as previously described [11] using the following cyclin D1 primer set: 5′-AAGCTGTGCATCTACACCGA-3′, and 5′-CTTGAGCTTGTTCACCAGGA-3′. Probe for cyclin D1 was 5′Fam-CCATTTGCAGCAGCTCCTCGG-3′Tamra. The primer pairs and Taqman probes for survivin, β-catenin, p53, p21, and β-actin have been published previously [11], [12], [13]. Eight microgram total RNA was separated on denatured agarose gel and transferred to Hybond N^+^ membrane. Northern blot analysis was performed using a random primer labeled [^32^P] cyclin D1 probe.

### [^3^H]-amino acid mixture incorporation assay

HepG2, HeLa, and PANC-1 cells labeled with [^3^H]-amino acid mixture were either treated with DCB-3503 or CHX, or left untreated as control. The incorporation of [^3^H]-amino acid into TCA-insoluble cellular components of each treatment was quantified using Beckman scintillation counter (Beckman Instruments, Fullerton, CA).

### [^35^S]-amino acid mixture incorporation assay

HepG2 cells were labeled with 50 µCi/ml [^35^S]-methionine/cysteine for 30 minutes before harvest. Incorporation of [^35^S]-methionine/cysteine was determined either by SDS-PAGE for protein profiling or by immunoprecipitation with cyclin D1 antibody.

### [^14^C]-thymidine and [^14^C]-uridine incorporation assay

HepG2 and PANC-1 cells labeled with [^14^C]-thymidine or [^14^C]-uridine were either treated with DCB-3503, CHX, hydroxyurea, actinomycin D, or left untreated as control [8]. The incorporation of [^14^C]-thymidine or [^14^C]-uridine into TCA-insoluble cellular components of each treatment was quantified using Beckman scintillation counter.

### *In vitro* transcription assay

Phagemid Bluescript SK- containing the cyclin D1 coding region was linerized by Hind III, and was used as the template for *in vitro* transcription. Uncapped Cyclin D1 mRNA was generated by MEGAscript kit containing T7 RNA polymerase (Ambion, Austin, TX). Capped cyclin D1 mRNA was generated by using a mMESSAGE mMACHINE high yield capped RNA transcription kit containing T7 RNA polymerase (Ambion). Polyadenylated cyclin D1 mRNA was generated by using a poly(A) tailing kit (Ambion). Luciferase encoding plasmids T3 luc and T3 luc (A) (poly A tail of 98 adenosines) were linearized by BamHI, and were used as the template for the following *in vitro* transcription. Uncapped luciferase mRNA with poly(A) tail was generated by MEGAscript kit containing T3 RNA polymerase (Ambion). Capped luciferase mRNA with and without poly(A) tail was generated by mMESSAGE mMACHINE high yield capped RNA transcription kit containing T3 RNA polymerase (Ambion). All the *in vitro* transcribed mRNAs were purified by MEGAclear kit (Ambion). The integrity of all the *in vitro* transcribed mRNAs was confirmed by denatured agarose gel electrophoresis. The purified mRNAs were used for *in vitro* translation experiments.

### *In vitro* translation using HeLa cell free system

The procedure for the extraction of HeLa cytosolic extracts for *in vitro* translation has been described previously [14]. The *in vitro* translation mixtures containing 50 ng/µl cyclin D1 mRNA were incubated at 30°C for 90 minutes. Translation products of cyclin D1, T3 luc, and T3 luc(A) mRNAs were resolved by SDS-PAGE followed by autoradiography, or luciferase assay, respectively.

### Luciferase assay

Luciferase activity was measured by luciferase assay kit (Promega) according to the manufacturer's instructions.

### Polysome profile analysis

Polysome profile analysis was performed essentially as described previously [15]. In brief, cytoplasmic extracts from control or drug treated cells were overlayed on the 10–50% sucrose gradients and centrifuged at 39,000 rpm for 80 minutes at 4°C using a SW 41Ti Beckman Coulter rotor (Beckman Coulter, Brea, CA). Fractions were collected with a continuous autodensi-flow density gradient fractionator (Labconco, Kansas City, MO). Total RNA from each fraction was isolated by Trizol (Invitrogen) method. Amount of specific mRNAs in each fraction was analyzed by real-time PCR.

## Results

### DCB-3503 suppresses the expression of a subset of proteins

The expression of cylcin D1, survivin, β-catenin, p53, and p21 were down-regulated by DCB-3503 in a time- and dose-dependent manner in HepG2 cells using Western blot analysis (Fig. 1B). In contrast, the expression of cyclin E and β-actin did not significantly change under the same conditions. Cyclin D1 was down-regulated as early as 15 minutes after treatment with 300 nM DCB-3503 (Fig. 1B left panel, compare Lane 2 and Lane 1), and its expression was suppressed extensively by as low as 50 nM DCB-3503 treatment for 2 hours (Fig. 1B right panel, compare Lane 2 and Lane 1). DCB-3503 induced down-regulation of cyclin D1, survivin, and β-catenin in the same fashion in HeLa cells (Fig. 1C). The down-regulatory effect of DCB-3503 on p53 and p21 in HeLa cells was difficult to evaluate due to the low endogenous level of these proteins (data not shown). The time- and dose-dependent suppression of cyclin D1 induced by DCB-3503 treatment was also observed in Huh7 and MCF-7 cells (Fig. 1D). Therefore, the down-regulatory effect of DCB-3503 on proto-oncoprotein cyclin D1 is not cell type-specific. This is in agreement with our previous findings that DCB-3503 down-regulated cyclin D1 in HepG2, and PANC-1 cells [6], [7].

### DCB-3503 does not decrease mRNA levels of the suppressed proteins

**Figure 2:**
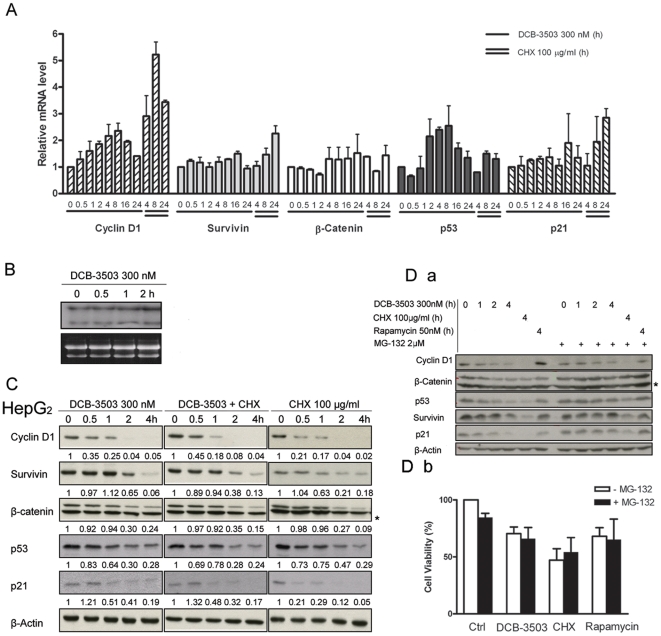
DCB-3503 did not decrease mRNA levels of cyclin D1, survivin, β-catenin, p53, and p21. **A.** HepG2 cells were treated with either DCB-3503 or CHX for the indicated time. mRNA levels of cyclin D1, survivin, β-catenin, p53, and p21 were analyzed by real-time RT-PCR using specific primers and probes and were normalized to that of β-actin. Values are depicted as mean ± S.D. from at least three separate experiments and are expressed relative to the basal mRNA level in the corresponding untreated control cells. **B.** mRNA level of cyclin D1 was analyzed by Northern blot under DCB-3503 treatment. Ribosomal RNA gel image on the bottom panel shows equal loading of total RNA. **C.** Expressions of cyclin D1, survivin, β-catenin, p53, and p21 under treatment of CHX, DCB-3503, and CHX and DCB-3503 in combination were examined by Western blot analysis in HepG2 cells. β-Actin blot was used as internal loading control. **D.** (***a***) 2 µM MG-132 reverses suppression of cyclin D1, survivin, β-catenin, p53, and p21 by DCB-3503 within 4-hour culture, (***b***) 2 µM MG-132 does not block cytotoxicity of DCB-3503 after 24-hour treatment. **B**, **C**, and **D** are representatives from at least three separate experiments. Numbers marked in **C** are normalized band intensity. (* Indicating non-specific band).

To analyze the underlying mechanism of DCB-3503 suppressed protein expression, we first assessed whether DCB-3503 could affect mRNA levels of these proteins using real-time PCR analysis. DCB-3503 caused a steady increase to about 2 fold in mRNA levels of cyclin D1 and p53 during the first 8-hour of treatment and then tapered off to the untreated control level by 24 hours (Fig. 2A). DCB-3503 exhibited little effect on mRNA levels of survivin, β-catenin, and p21 through out the treatment (Fig. 2A). The increase in mRNA level could be due to the compensatory regulation of cyclin D1 and p53 proteins. In contrast, CHX significantly increased mRNA levels of cyclin D1 and p21 after 24 hours treatment, but did not alter mRNA levels of other proteins studied (Fig. 2A). Northern blot analysis revealed that cyclin D1 mRNA level was increased by DCB-3503 after 2-hour treatment (Fig. 2B). The 4.8 kb and 1.7 kb RNA transcripts of cyclin D1, which have the same coding sequence but differ in polyadenylation [16], remained unchanged after treatment with DCB-3503. Thus, DCB-3503 suppression of protein accumulation cannot be due to the decrease of mRNA levels. However, we cannot rule out the possibility that the unchanged transcripts of cyclin D1 mRNA are due to the compensation of the inhibitory effect of DCB-3503 on cyclin D1 expression.

### DCB-3503 dose not increase the degradation of proteins with short half-lives

We next examined whether DCB-3503 treatment could induce degradation of cyclin D1, survivin, β-catenin, p53, and p21. CHX blocks peptidyl transferase activity in eukaryotic ribosomes, and it was used to stop protein synthesis in eukaryotic cells for the estimation of the half-life. We found that the half-life of cyclin D1 in HepG2 cells is relatively short (<0.5 hour) as compared to that of β-actin (>4 hours, Fig. 2C, right panel). The half-lives of other proteins in the presence of CHX was determined to be in the order of survivin ≥β-catenin > p53 > cyclin D1 ≥ p21. We next assessed whether the combined treatment of CHX and DCB-3503 could enhance the suppression of the cell regulatory proteins we examined, compared to CHX treatment alone. The intensities of the bands of these proteins were quantified using a densitometer, and normalized to that of β-actin and marked below each band (Fig. 2C). When CHX was present, there was no detectable difference in the kinetics of decay of cyclin D1, survivin, β-catenin, p53, and p21 in the presence or absence of DCB-3503 (Fig. 2C). Pre-exposure of HepG2 cells to 2 µM MG-132, a proteasome inhibitor, following the addition of DCB-3503 reversed the suppression of cyclin D1, survivin, β-catenin, p53, and p21 by DCB-3503 treatment within 4 hours (Fig. 2D *a*). However, MG-132 at 2 µM cannot abolish the cytotoxicity of DCB-3503 after 24-hour treatment (Fig. 2D *b*). Higher concentration of MG-132 (10 µM) caused cell death in the presence or absence of DCB-3503 with overnight culture (data not shown). Taken together, these results indicate that DCB-3503 does not increase the rate of degradation of this subset of proteins.

### DCB-3503 inhibits amino acid incorporation in a time- and dose-dependent manner

**Figure 3:**
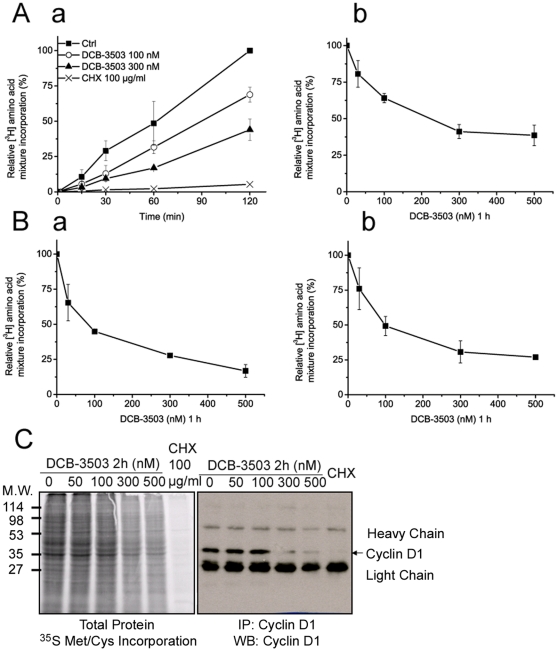
DCB-3503 inhibited incorporation of amino acid in a time- and dose-dependent manner. **A.** DCB-3503 inhibited [^3^H]-amino acid incorporation in both time- (***a***) and dose-dependent (***b***) manner in HepG2 cells. **B.** DCB-3503 inhibited [^3^H]-amino acid incorporation in HeLa cells (***a***) and PANC-1 cells (***b***) in a dose-dependent fashion. **A** and **B** results from three separate experiments are presented as mean ± S.D. **C.** Inhibitory effect of DCB-3503 on global protein synthesis and cyclin D1 protein synthesis assessed by [^35^S]-methionine/cysteine incorporation. This is a representative from three separate experiments.

The above results indicated that DCB-3503 suppresses cell regulatory proteins at the translational level. We therefore measured global changes of protein synthesis in response to DCB-3503 treatment. A time course of [^3^H]-amino acid incorporation into proteins is affected by DCB-3503 as shown in Fig. 3A. At 100 nM, DCB-3503 inhibited [^3^H]-amino acid incorporation as early as by 15 minutes of treatment (Fig. 3A *a*). This inhibition was DCB-3503 dose-dependent but not cell type specific, as observed in HeLa (Fig. 3B *a*) and PANC-1 cells (Fig. 3B *b*). DCB-3503 treatment inhibited [^3^H]-amino acid incorporation by 50% at 100 nM, whereas 100 µg/ml of CHX totally blocked it (Fig. 3A *a*). This could imply an enhanced susceptibility to DCB-3503 mediated inhibition for a subset of proteins. In addition, suppression of global protein synthesis by DCB-3503 was revealed by [^35^S]-methionine/cysteine incorporation assay in HepG2 cells (Fig. 3C, left panel). The amount of newly synthesized cyclin D1 protein in DCB-3503 treated HepG2 cells was assessed by immunoprecipitation with an anti-cyclin D1 antibody of [^35^S]-labeled proteins. There was a reduction in the amount of radioactivity in a major immunoprecipitable polypeptide that corresponds to cyclin D1 at 300 nM as established by immunoblotting (Fig. 3C, right panels). DCB-3503 inhibited cyclin D1 and total protein synthesis with similar efficiency as indicated by the quantitative analysis of [^35^S] incorporation (Fig. S1E). These results indicate that DCB-3503 inhibits global protein synthesis, which has preferential effect on proteins with short half-life, like cyclin D1.

### DCB-3503 preferentially inhibits [^14^C]-thymidine incorporation, but not [^14^C]-uridine incorporation, in a time- and dose-dependent manner

**Figure 4:**
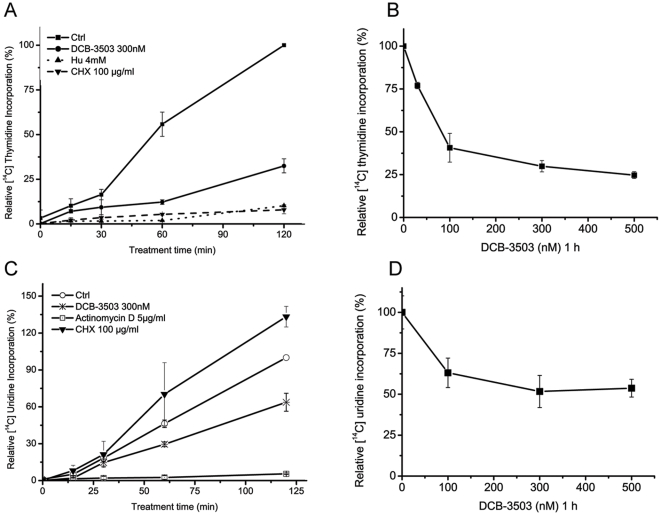
DCB-3503 preferentially inhibited [^14^C]-thymidine incorporation in a time- and dose-dependent manner, but not [^14^C]-uridine incorporation. **A.** and **B.** The inhibitory effect of DCB-3503 on [^14^C]-thymidine incorporation was time-dependent and dose-dependent in HepG2 cells. (**C**) and (**D**) DCB-3503 showed less than 50% inhibition on [^14^C]-uridine incorporation in either HepG2 cells followed by different time and dose treatment. Hydroxyurea (Hu) and actinomycin D were used as positive controls. Values are depicted as mean ± S.D. from at least three separate experiments.

In addition to protein synthesis, nucleic acid synthesis is inhibited by tylocrebrine [17]. We investigated DNA and RNA synthesis by monitoring [^14^C]-thymidine and [^14^C]-uridine incorporation in HepG2 cells. Time course studies indicated that 300 nM DCB-3503 began to show inhibitory effect on [^14^C]-thymidine incorporation into DNA within 30 minutes of treatment (Fig. 4A). In addition, there was dose-dependent down-regulation of [^14^C]-thymidine incorporation into DNA by DCB-3503 treatment in both PANC-1 and HepG2 cells (Fig. 4B and supplemental Fig. S1). DCB-3503 caused less than 50% inhibitory effect on [^14^C]-uridine incorporation into RNA at 300 nM after 2-hour treatment (Fig. 4C and D). Increasing concentrations of DCB-3503 beyond 300 nM did not further exaggerate the inhibition of [^14^C]-uridine incorporation (Fig. 4D). CHX did not show inhibitory effect on [^14^C]-uridine incorporation under the same conditions (Fig. 4C). Similar results were obtained in PANC-1 cells (Supplemental Fig. S1).

### DCB-3503 inhibits translation of cyclin D1 mRNA in HeLa cell extracts at high concentrations

**Figure 5:**
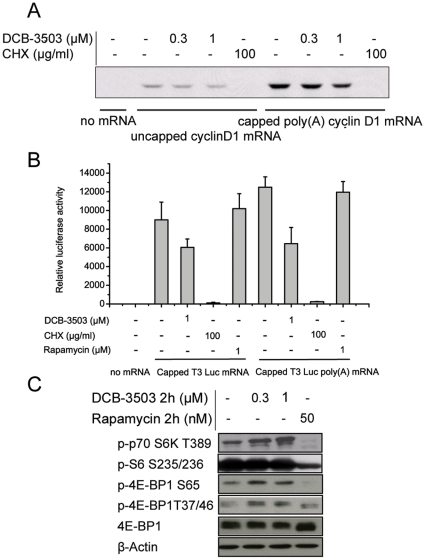
DCB-3503-mediated inhibition of protein synthesis differs from that of CHX and rapamycin. **A.** Effects of DCB-3503 and CHX on translation of uncapped and capped poly(A) tail cyclin D1 mRNAs in HeLa cell free lysate system. **B.** Effects of DCB-3503, CHX, and rapamycin on translation of capped luciferase mRNAs with or without 3'poly(A) tail in HeLa cell free lysate system. Values are depicted as mean ± S.D. from at least three separate experiments. **C.** Western blot analysis of phosphorylated p70 S6Kinase T^389^, S6 S^235/236^, phosphorylated 4EBP1 S^65^ and T^37/46^ under DCB-3503 and rapamycin treatment for 2 hours in HepG2 cells. 4EBP1 and β-Actin blots were used as internal loading controls. **A** and **C** are representatives from three separate experiments.

Since DCB-3503 treatment inhibited incorporation of amino acid and cyclin D1 synthesis in cell culture, we investigated whether DCB-3503 treatment could inhibit translation of cyclin D1 mRNA *in vitro*. Attempts to set up *in vitro* translation system using PANC-1 cells (referred to originally established methods with HeLa [18], [19]) and HepG2 cells (methods adapted as published previously [20], [21]) to translate exogenously mRNA were not successful. We therefore established HeLa cell extract *in vitro* translation system [14]. This system was reported to recapitulate synergistic stimulation of translation by the terminal mRNA structures, the 3′ poly(A) tail and the 5′ cap [14]. Consistent with previous report, capped polyadenylated cyclin D1 mRNA had significantly higher translation efficiency in comparison to that of uncapped non-polyadenylated cyclin D1 mRNA (Fig. 5A, compare lane 6 to lane 2). Three hundred nanomoles of DCB-3503 inhibited less than 50% of translation of either uncapped (Fig. 5A, compare lane 3 to lane 2) or capped cyclin D1 mRNA compared to untreated control level (Fig. 5A, compare lane 7 and lane 6). However, 1 µM of DCB-3503 treatment markedly inhibited translation of both uncapped and capped cyclin D1 mRNA (Fig. 5A, compare lane 4 to lane 2, and lane 8 to lane 6). By comparison, CHX completely blocked both capped and uncapped cyclin D1 mRNA translation *in vitro* (Fig. 5A, compare lane 5 to lane 2, and lane 9 to lane 6). Similar results were obtained using T3 luciferase mRNA as a model (discussed below).

### DCB-3503 acts differently from CHX and rapamycin

Translation efficiency is positively regulated by the 5′ cap structure and the 3′ poly(A) tail. We therefore used capped luciferase mRNA with or without poly(A) tail to program HeLa cell-free extracts. The addition of 1 µM DCB-3503 produced about 50% inhibitory effect on translation of both non-polyadenylated and polyadenylated luciferase mRNAs (Fig. 5B). CHX completely blocked translation of T3 luciferase mRNA *in vitro*. The mTOR inhibitor, rapamycin, had no inhibitory effect on translation of either type of mRNA under the same conditions. Western blot results showed DCB-3503 elevated phosphorylations of p70 S6 kinase, 4E-BP1 at Ser^65^ and Thr^37/46^ sites, and had no effect on phosphorylation of S6 ribosomal after 2-hour treatment in HepG2 cells (Fig. 5C). In contrast, rapamycin completely inhibited phosphorylation of p70 S6 kinase (Thr^389^) and reduced phosphorylated S6 ribosomal protein (Ser^235/236^) level to about 50% of untreated control under the same condition (Fig. 5C). Rapamycin only suppressed phosphorylation of 4E-BP1 at Ser^65^, which can inhibit cap-dependent translation downstream of mTOR by inhibiting the rate-limiting translational initiator eIF4E [22], [23], [24]. These results indicate that the action of DCB-3503 is different from that of CHX and rapamycin.

### DCB-3503 induces changes in polysome profile and mRNA distribution

**Figure 6:**
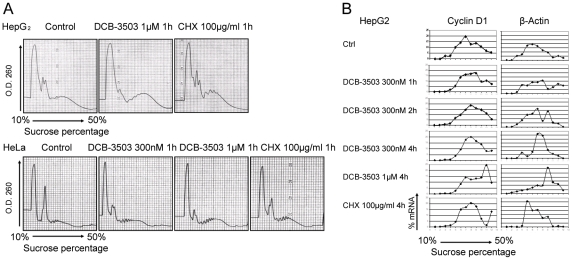
DCB-3503 inhibited the elongation step of translation. **A.** DCB-3503 treatment induced accumulation of polysomes in HepG2 and HeLa cells after 1-hour treatment. **B.** DCB-3503 induced accumulation of mRNAs of cyclin D1 and β-Actin in polysomal fractions in the sucrose gradient in HepG2 cells. **A** and **B** were representatives from three separate experiments with similar results.

To further characterize the effects of DCB-3503 on translation, we compared polysome profiles of DCB-3503 or CHX-treated HepG2 and HeLa cells to untreated control cells. DCB-3503 increased the abundance of polysomes at the expense of monosomes, with heavy polysomes being relatively more affected than light polysomes (Fig. 6A). Such an effect on the polysome profile is a hallmark of inhibition of elongation [25]. CHX treatment stabilized polysomes in both HepG2 and HeLa cells (Fig. 6A). We then analyzed distribution of mRNAs of cyclin D1, survivin, β-catenin, and β-actin in the sucrose gradient fractions from HepG2 cells using real-time PCR. DCB-3503 induced sedimentation shift of all these mRNAs to the non-translation proficient polysome fractions of the gradient in a time- and dose-dependent manner (Fig. 6B and Fig. S2). The increased accumulation of cyclin D1, survivin, β-catenin, and β-actin mRNAs in the polysome fractions is consistent with the inhibition of the elongation step of translation by DCB-3503. This result is in line with previous report that tylophorine analogs prevent break down of polysomes in *Saccharomyces cerevisiae* [3].

## Discussion

This study demonstrates that cyclin D1 protein could be down-regulated as early as 15 minutes after DCB-3503 treatment (Fig. 1). In contrast, availability of cyclin D1 mRNA remain unchanged under similar condition (Fig. 2A and B). We also found that DCB-3503 treatment suppressed the expression of cyclin D1, survivin, β-catenin, p53, and p21. Combined treatment of DCB-3503 and CHX did not further decrease the half-lives of these proteins as compared to the treatment with CHX alone (Fig. 2C). Treatment of DCB-3503 inhibited the incorporation of [^3^H]- or [^35^S]-amino acid into proteins as measured in HepG2, HeLa, and PANC-1 cells in a time- and dose-dependent manner (Fig. 3). In addition, we observed that DCB-3503 preferentially suppressed the rate of [^14^C]-thymidine incorporation into DNA (Fig. 4A), but this could be a secondary event following the down-regulation of a subset of cellular proteins involved in DNA synthesis. Xi et al reported an alternative possibility that tylophorine analogs can interact with bulged DNA directly, and then interfere with DNA synthesis [26]. However, we were unable to demonstrate that DCB-3503 interacts with DNA using ethidium displacement analysis (data not shown).

A variety of antitumor agents suppress transcription and/or translation of cyclin D1; these include retinoic acid, rapamycin, LY294002, wortmannin, herbimycin A, and geldanamycin [27], [28]. Additionally, inhibitors of translational elongation like homoharringtonine, bruceantin, and didemnin B, can also inhibit expression of cyclin D1 [29]. These compounds down-regulate cyclin D1 expression through different mechanisms. Retinoic acid induces proteasomal degradation of cyclin D1 and exhibits no effect on cyclin D1 mRNA level. GSK3 plays a key role in retinoid regulation of cyclin D1 [28]. Rapamycin, a well-known mTOR inhibitor, suppresses initiation of cyclin D1 mRNA translation through inhibition of the mTOR-ribosomal p70 S6 kinase and mTOR-eukaryotic initiation factor 4E-binding protein-1 (eIF4E) pathways. In addition, rapamycin-stimulated activation of GSK3β leads to a decrease in the half-life of cyclin D1 and induction of cyclin D1 proteolysis [27]. Differential inhibition of cyclin D1 expression by PI3K inhibitors, LY294002, and wortmannin, is mediated through differential inhibition of p70 S6 kinase [30]. Herbimycin A, a potent tyrosine kinase inhibitor, down-regulates cyclin D1 protein by suppressing synthesis of its mRNA [31]. Geldanamycin, a potent heat shock protein 90 (HSP90) inhibitor, can induce cyclin D1 down-regulation through suppression of mTOR signaling pathway by disrupting the *in vivo* binding of HSP90 with mTOR raptor [32]. Homoharringtonine and bruceantin inhibit polysome chain elongation on poly(U)-programmed 80S ribosomes, as assayed by polysome profiling [33], [34]. Didemnin B suppressed translation through binding to the eukaryotic elongation factor 1α in GTP-dependent manner [35]. The action of DCB-3503 on inhibition of protein translation is distinct from that of the above-mentioned compounds, and is apparently operating at the elongation phase of translation (Fig. 6).

Huang et al reported that 100 µM of tylocrebrine inhibited protein synthesis during chain elongation [17]; Grant et al reported that 130 µM of cryptopeurine inhibited protein synthesis in the translocation phase [36]. These concentrations were far above the relevant concentration we studied (100 nM, 300 nM or maximum 1 µM, Fig. 5 and 6). The structures of the above two tylophorine analogs are different from DCB-3503. These structure analogs of tylophorine may not be functional analogs, as we demonstrated previously [8]. The high cytotoxicity of DCB-3503 may also be due to its inhibition of elongation phase in translation (Fig. 6). Translation elongation inhibitors can sensitize chemotherapeutic agents [29], [37], [38]. Additionally, inhibition on translation elongation can overcome drug resistance [39], [40]. One possibility is that the proto-oncoproteins and pro-survival factors exhibit higher translation rates in cancer cells, and inhibition of all mRNAs elongation will turn out to have more profound effect on mRNAs of these proteins [41]. Alternatively, proto-oncoproteins and pro-survival factors have relatively short half-lives, and are more likely to be depleted by inhibition of elongation [29], [42].

We observed partial inhibitory effect on translation with 1 µM DCB-3503 in Hela cell extracts system (Fig. 5A), which is less effective dosage on cell based studies (Fig. 1). Intracellular regulators that control elongation may be only partially operative in *in vitro* translation systems [43]. Such factors can be elongation factors, or recently actively studied miRNAs. Phosphorylation status of initiation factors and components of signaling pathways may vary in *in vitro* systems and in intact cells. In cell free systems, eukaryotic translation initiation factor 2α (eIF2α) and eIF2α kinases, and many other kinases become phosphorylated due to the presence of essential supplements, such as ATP and creatine phosphate [44]. Phosphorylation of eIF2α may introduce a new limiting step in the translation process at the level of initiation [45]. Thus, DCB-3503-induced decrease in the rate of polypeptide chain elongation may not be fully reflected in the rate of protein synthesis *in vitro*, in contrast to the situation *in vivo*. miRNAs are small noncoding RNAs that regulate the stability and/or translation of mRNAs [46]. Preliminary studies of miRNA profiling showed that DCB-3503 altered the homeostasis of a number of miRNAs after 4-hour treatment (unpublished data). The possibility that the regulated miRNA homeostasis is a cause or consequence of inhibition of protein synthesis is currently under investigation.

In summary, our results indicate that DCB-3503 inhibits protein synthesis by reducing the rate of polypeptide chain elongation. The global inhibition effect on protein synthesis, however, could be expected to suppress proteins with a shorter half-life more profoundly than proteins with a longer half-life. The immediate and rapid changes of these proteins regulated by translation are important in physiological conditions like apoptosis and cellular stress. Such proteins could include cyclin D1 or co-activators regulating NF-κB mediated transcription. The half-lives and the roles of these proteins down-regulated by DCB-3503 in different cells or tissues could be different. Long-term suppression of protein synthesis could lose the selectivity. This can explain why DCB-3503 has therapeutic activity against cancer cell growth, lupus, and arthritis in mice when it is given every three days, as we reported previously [5], [6], [47], [48]. Therapeutic index of DCB-3503 against cancer cells *in vivo* may be lost when it is given daily. The effectiveness of this class of analogs for the treatment of disease will be highly schedule-dependent. The mechanism of inhibition of protein synthesis by DCB-3503 is likely to be distinct from the known inhibitors of protein synthesis. Unraveling this mechanism may open a window for new therapeutic opportunities for the treatment of human cancers, including hepatocellular carcinoma. DCB-3503 may be used in combination treatment of malignant cancers with therapeutic agents that show different mechanisms of action. It can also be used in the treatment of resistance phenotypes characterized by MDR-1 and MRP-1 overexpression.

## Supporting Information

Figure S1 DCB-3503 inhibited [14C]-thymidine incorporation in a time- and dose-dependent manner, but not [14C]-uridine incorporation in PANC-1 cells. The inhibitory effect of DCB-3503 on [14C]-thymidine incorporation was time-dependent (A) and dose-dependent (B) in PANC-1 cells. DCB-3503 showed less than 50% inhibition on [14C]-uridine incorporation in either PANC-1 cells followed by different time (C) or dose treatment (D). E. Normalized relative incorporation of [35S]-methionine/cysteine into total protein and cyclin D1 presented in Figure 3C through three separate experiments and presented as mean ± S.D.
(6.23 MB TIF)

Figure S2 Effects of DCB-3503 on mRNA distribution of cyclin D1, survivin, β-catenin, and β-actin in fractions HepG2 cells obtained from sucrose gradient were quantitated by real-time RT PCR.
(6.43 MB TIF)
