# Supplementary figures and images for: DCB-3503, a Tylophorine Analog, Inhibits Protein Synthesis through a Novel Mechanism
Source: PLoS One. 2010 Jul 15;5(7):e11607. doi: 10.1371/journal.pone.0011607 (PMC2904705; doi:10.1371/journal.pone.0011607)

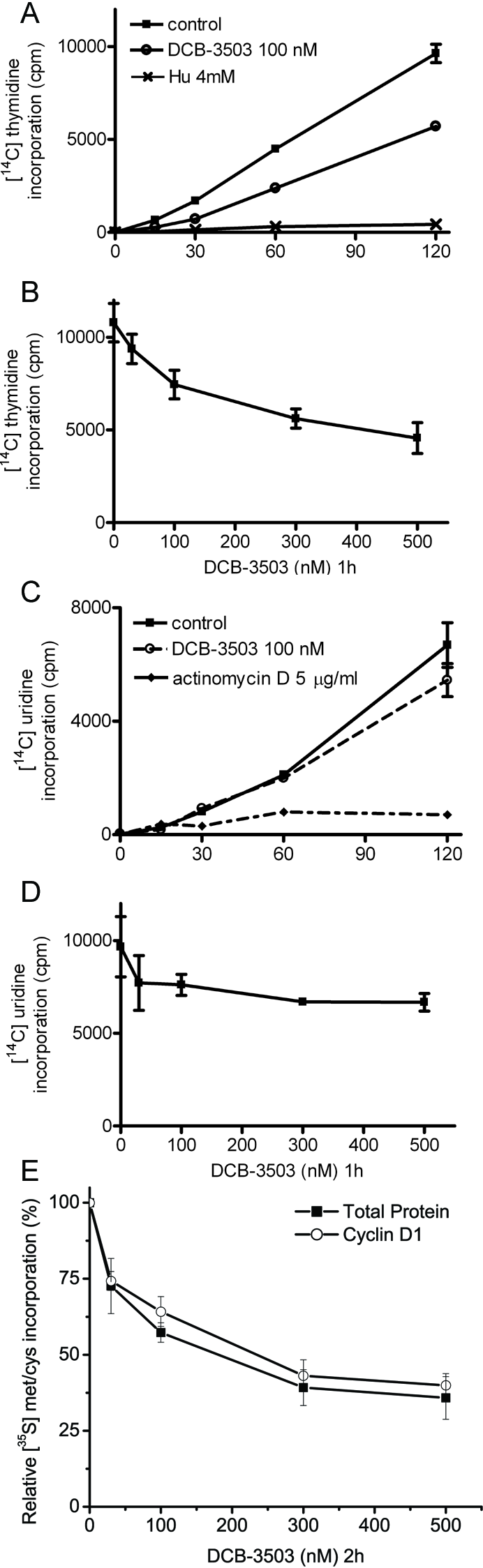

Supplement: Figure S1 — DCB-3503 inhibited [14C]-thymidine incorporation in a time- and dose-dependent manner, but not [14C]-uridine incorporation in PANC-1 cells. The inhibitory effect of DCB-3503 on [14C]-thymidine incorporation was time-dependent (A) and dose-dependent (B) in PANC-1 cells. DCB-3503 showed less than 50% inhibition on [14C]-uridine incorporation in either PANC-1 cells followed by different time (C) or dose treatment (D). E. Normalized relative incorporation of [35S]-methionine/cysteine into total protein and cyclin D1 presented in Figure 3C through three separate experiments and presented as mean ± S.D. (6.23 MB TIF) [file pone.0011607.s001.tif]

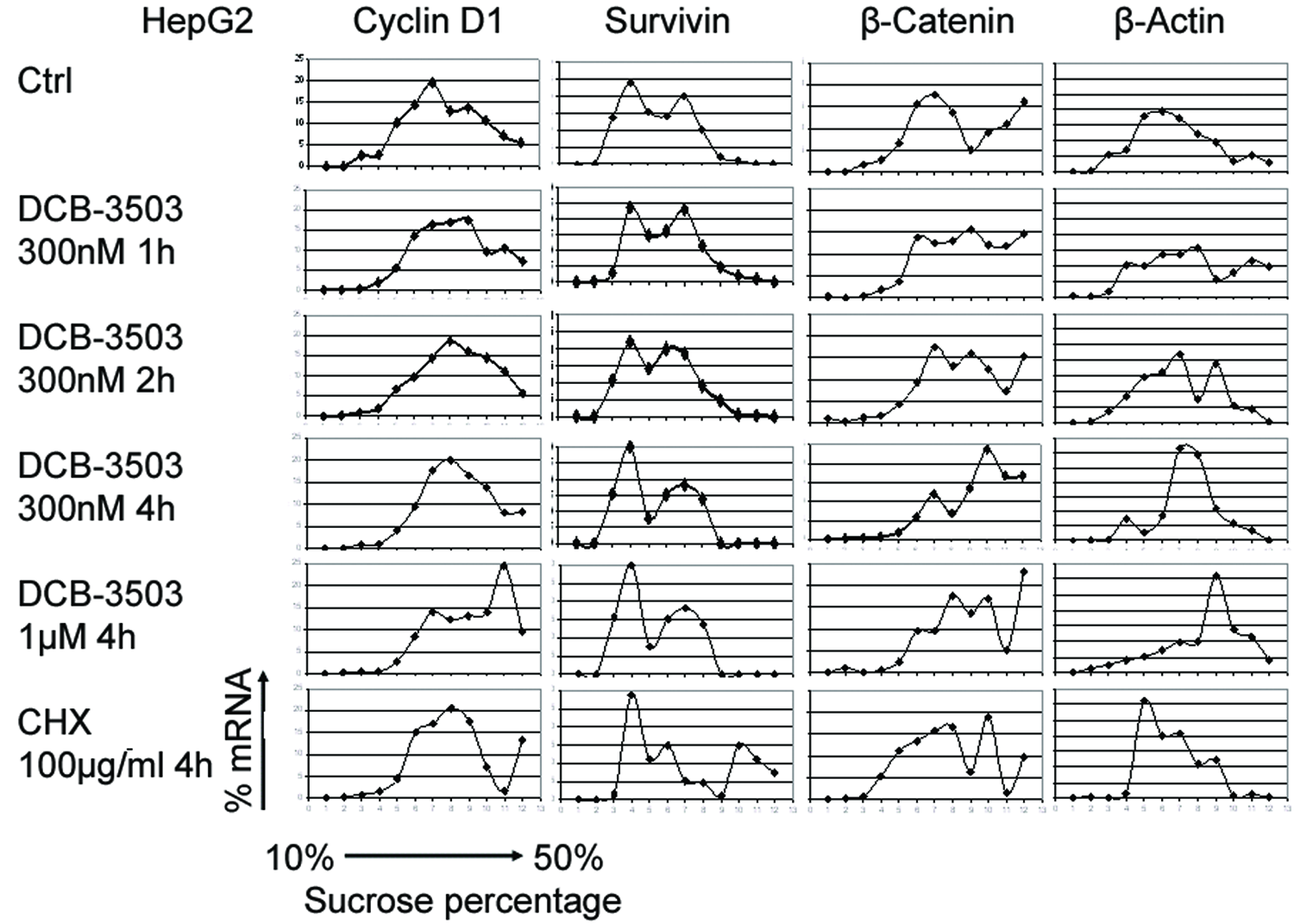

Supplement: Figure S2 — Effects of DCB-3503 on mRNA distribution of cyclin D1, survivin, β-catenin, and β-actin in fractions HepG2 cells obtained from sucrose gradient were quantitated by real-time RT PCR. (6.43 MB TIF) [file pone.0011607.s002.tif]
